# Is Trabecular Bone Score Valuable in Bone Microstructure Assessment after Gastric Bypass in Women with Morbid Obesity?

**DOI:** 10.3390/nu9121314

**Published:** 2017-12-02

**Authors:** Agustina Pia Marengo, Fernando Guerrero Pérez, Luis San Martín, Rosa Monseny, Anna Casajoana, Rocio Valera, Nuria Virgili, Andreu Simó Servat, Albert Prats, Carmen Gómez-Vaquero, Nuria Vilarrasa

**Affiliations:** 1 Department of Endocrinology and Nutrition, Hospital Universitari de Bellvitge-IDIBELL, c/ FeixaLlarga s/n, L’Hospitalet de Llobregat, 08907 Barcelona, Spain; agusmarengo@hotmail.com (A.P.M.); ferguepe@hotmail.com (F.G.P.); lsanmartin@bellvitgehospital.cat (L.S.M.); ro.valera83@gmail.com (R.V.); mvirgili@bellvitgehospital.cat (N.V.); andreusimoservat@gmail.com (A.S.S.); 2 Clinical Nutrition Unit, Hospital Universitari de Bellvitge-IDIBELL, c/ FeixaLlarga s/n, L’Hospitalet de Llobregat, 08907 Barcelona, Spain; monseny@bellvitgehospital.cat; 3 Bariatric Surgery Unit, Hospital Universitari de Bellvitge-IDIBELL, c/ Feixa Llarga s/n, L’Hospitalet de Llobregat, 08907 Barcelona, Spain; tesalonica@gmail.com; 4 Department of Rheumatology, Hospital Universitari de Bellvitge-IDIBELL, c/ FeixaLlarga s/n, L’Hospitalet de Llobregat, 08907 Barcelona, Spain; aprats@bellvitgehospital.cat; 5 CIBERDEM-CIBER de Diabetes y Enfermedades Metabólicas Asociadas, Instituto de Salud Carlos III, Madrid, Spain

**Keywords:** bone mineral density, osteoporosis, body composition

## Abstract

Introduction: The effects of bariatric surgery on skeletal health raise many concerns. Trabecular bone score (TBS) is obtained through the analysis of lumbar spine dual X-ray absorptiometry (DXA) images and allows an indirect assessment of skeletal microarchitecture (MA). The aim of our study was to evaluate the changes in bone mineral density (BMD) and alterations in bone microarchitecture assessed by TBS in morbidly obese women undergoing Roux-en-Y gastric bypass (RYGB), over a three-year follow-up. Material/Methods: A prospective study of 38 morbidly obese white women, aged 46.3 ± 8.2 years, undergoing RYGB was conducted. Biochemical analyses and DXA scans with TBS evaluation were performed before and at one year and three years after surgery. Results: Patients showed normal calcium and phosphorus plasma concentrations throughout the study. However, 25-hydroxyvitamin D (25(OH)D_3_) decreased, and 71% of patients had a vitamin D deficiency at three years. BMD at femoral neck and lumbar spine (LSBMD) significantly decreased 13.53 ± 5.42% and 6.03 ± 6.79%, respectively, during the three-year follow-up; however *Z*-score values remained above those for women of the same age. TBS was within normal ranges at one and three years (1.431 ± 106 and 1.413 ± 85, respectively), and at the end of the study, 73.7% of patients had normal bone MA. TBS at three years correlated inversely with age (*r* = −0.41, *p* = 0.010), body fat (*r* = −0.465, *p* = 0.004) and greater body fat deposited in trunk (*r* = −0.48, *p* = 0.004), and positively with LSBMD (*r* = 0.433, *p* = 0.007), fat mass loss (*r* = 0.438, *p* = 0.007) and lean mass loss (*r* = 0.432, *p* = 0.008). In the regression analysis, TBS remained associated with body fat (*β* = −0.625, *p* = 0.031; *R*^2^ = 0.47). The fracture risk, calculated by FRAX^®^ (University of Sheffield, Sheffield, UK), with and without adjustment by TBS, was low. Conclusion: Women undergoing RYGB in the mid-term have a preserved bone MA, assessed by TBS.

## 1. Introduction

Although bariatric surgery is an effective treatment for morbidly obese patients, that leads to improvement or even resolution of associated comorbidities, it is not immune from nutritional and metabolic complications. Due to this, concerns have been raised over its adverse effects on skeletal health, as increased bone loss and fragility have been described after bariatric surgery that might lead to increased bone fractures [1]. Few reviews and meta-analysis have analyzed BMD changes after bariatric surgery in the short-term and most of them have reported a significant decrease, mainly at the femoral neck [2,3,4,5,6,7,8]. However, BMD after obesity surgery was not found to be different at either the femoral neck or lumbar spine in cross-sectional studies comparing BMI matched controls [9,10,11]. Also, contradictory results have been described regarding increased fracture risk after bariatric surgery [12,13,14,15,16]. Bone mineral density (BMD), measured by dual-energy X-ray absorptiometry (DXA), is a major determinant of bone strength and fracture risk. However, most individuals with a fragility fracture will have BMD in the osteopenic or even normal range, indicating the importance of bone quality with preserved architecture over bone quantity [17]. In the search for a new and non-invasive tool for assessing skeletal microarchitecture (MA), trabecular bone score (TBS) has been developed. TBS is obtained through the analysis of two-dimensional DXA projection images of lumbar spine using specific hardware and software [18,19]. Higher TBS values indicate stronger microarchitecture, whereas lower scores reflect poor bone quality with a greater susceptibility to fracture [18]. It is expected that TBS could complement BMD in the prediction of risk fracture and has therefore been incorporated into the FRAX^®^ algorithm [20]. TBS values are accurate only for patients with a body mass index (BMI) in the range 15 to 35 kg/m^2^. The applicability of this method in the assessment of bone MA and fracture risk after bariatric surgery may be useful. However, data in this population so far are limited to only one previous study, in which TBS was analyzed in a cohort of premenopausal women and men undergoing different types of bariatric techniques (Roux-en-Y gastric bypass (RYGB) and Sleeve Gastrectomy (SG)) with a 2-year follow-up. TBS decreased during the first 6 months and then remained unchanged in patients randomized to supplementation with calcium, vitamin D, proteins and physical exercise prescription. 

Against this background, the aim of our study was to evaluate changes in BMD parameters and the presence of alterations in bone MA assessed by TBS in a homogeneous cohort of morbidly obese women undergoing RYGB, over a three-year follow-up. We also analyzed the influence of body composition parameters on TBS values and calculated the fracture risk adjusted by this score, in order to obtain a more accurate assessment of major osteoporotic and hip fracture risk in this population.

## 2. Material and Methods

Forty-five consecutive morbidly obese women were recruited over a period of 18 months from the Endocrinology Clinic of the Hospital de Bellvitge (Barcelona, Spain). The inclusion criteria were women 18–60 years-old, BMI above 40 kg/m^2^ or 35 kg/m^2^ with associated obesity comorbidities, candidates in the waiting list for gastric bypass surgery, presence of normal calcium and phosphorous concentrations at the initial evaluation. The primary exclusion criteria were an acute major cardiovascular event in the previous six months, recent or ongoing infection, history of cancer disease, or treatment with anti-inflammatory drugs. Women with known osteoporosis, who were taking any medications, including hormone replacement therapy, or had a disease state known to influence calcium or bone metabolism, were also excluded. Forty morbidly obese women were selected for the study, but two women from the database could not be contacted for the three-year evaluation. Therefore, thirty-eight morbidly obese white women were re-evaluated 3 years after surgery and were finally included in the study. 

All participants had signed a written consent to be included in the study, which was approved by the research ethics board of our hospital. 

Patients underwent a RYGB operation by laparoscopy [21]. In brief, a small gastric upper pouch (30 mL) was created along the lesser curvature of the stomach. The length of the biliopancreatic limb was 60 cm and that of the alimentary limb was 120 cm. After RYGB, 800 IU of vitamin D and 1200 mg of calcium phosphate were prescribed as an oral supplement in all patients, in addition to a multivitamin pill twice daily. 

Pre and postoperative anthropometric measurements, BMD scanning using DXA were made, and blood samples were collected prior to surgery, and at 12 months and three years after surgery. 

### 2.1. Anthropometrical Measurements

Anthropometric measurements included weight, height, waist and hip circumference. BMI and waist to hip ratio were calculated. Obesity was classified according to the World Health Organization (WHO) criteria [22].

BMD (g/cm^2^) was measured at lumbar spine (LS) L2-L4 and proximal hip by DXA (Hologic QDR 4500; Hologic Inc., Waltham, MA, USA) in the Bone Densitometry Unit of the Rheumatology Service. Calibration with a lumbar spine phantom was performed daily. The *T*-score (number of standard deviations away from the mean BMD of young normal subjects of the same sex) and the Z-score (number of standard deviations away from the mean BMD of age and sex matched normal subjects) were established by comparison with data from the study of BMD in a Spanish population, performed by the Multicenter Research Project on Osteoporosis [23]. Osteopenia (*T*-score between −1.0 and −2.5) and osteoporosis (*T*-score below −2.5) were defined, according to the WHO criteria [24].

Lumbar spine DXA scans were recovered from the densitometer database and TBS was calculated for this study. The following normal range for TBS values was used, as proposed by the manufacturers MedImaps: A TBS ≥ 1.350 was considered to be normal; a TBS between 1.200 and 1.350 was considered to be consistent with partially degraded MA; and a TBS ≤ 1.200 was defined as degraded MA [25].

TBS values are accurate only for patients with BMIs in the range 15 to 35 kg/m^2^ and manufacturers do not recommend the interpretation of TBS in patients out of this range of BMI. Therefore, results will be presented as “All TBS” (including all patients regardless of BMI) and “Strict TBS” (including only patients with BMI < 35 kg/m^2^). In general, TBS in very obese patients seems to be underestimated.

The classic fracture risk assessment system, FRAX^®^, and the algorithm, FRAX corrected by TBS were used to evaluate the 10-year probability of fracture. The output obtained is a 10-year absolute probability of hip fracture and the probability of a major osteoporotic fracture (clinical spine, forearm, hip or shoulder fracture) [20,26].

Body composition (total percentage fat mass and total lean mass) was assessed by DXA. The scanning was performed with the patient in underwear, after all metallic artefacts were withdrawn, whole body scanning lasted 7 min; and body-fat distribution was measured using the body adiposity index. 

The same material and the same DXA device were used for anthropometrical and body composition measurements during the follow-up.

### 2.2. Analytical Methods

Blood samples were drawn from each subject between 8 and 9 a.m., before breakfast, after an overnight rest. All samples were stored at −80 °C until analytical measurements were performed.

25-hydroxyvitamin D (25(OH)D_3_) concentrations were determined using a radioimmunoassay (DiaSorin, Stillwater, MN, USA). A 25(OH)D_3_ deficiency was considered when their concentrations were determined to be lower than 50 nmol/L [27]. Intact serum parathyroid hormone (PTH) was measured by a two-site immunoradiometric assay (Diagnostic System Laboratories, Webster, TX, USA); intra and interassay coefficients of variation were 10% and 11%, respectively. The normal range for our laboratory was 1.6–6.9 pmol/L.

### 2.3. Statistical Analysis

Based on preliminary data, the study was designed to detect a TBS difference greater than 10%, before and after surgery, with a power of 80% and an α risk of 0.05 in a two-sided test. The standard deviation was assumed to be 0.15. A minimum of 17 subjects was necessary, assuming an anticipated a drop-out rate of 5%. Descriptive statistics are presented as mean ± standard deviation for variables with a normal distribution (Kolmogorov–Smirnov *p*-value > 0.05) and median (interquartile range) for non-normally distributed variables. The student´s *t*-test and analysis of variance (ANOVA) were used to compare normal distributed variables and the Wilcoxon test was used for non-normally distributed variables. Relationships among variables were tested by Pearson´s test in normally distributed variables and Spearman´s correlation coefficient in non-normally distributed variables, respectively. BMD changes were calculated using the formula: ((initial BMD − final BMD)/initial BMD × 100). A comparison between BMD values during follow-up was done using repeated measurements with student’s *t*-tests. A univariate analysis was performed to study the relationship of the final TBS with age, anthropometry, body composition parameters (lean mass, body fat percentage and distribution), BMD and also with changes in the previous parameters. A multiple regression analysis was performed to identify variables associated with TBS at the three-year evaluation point. In the model, the variables, age, lean mass, fat mass, PTH and vitamin D concentrations and their changes from baseline were included, respectively. In the multivariate analysis, colinearity among variables was sorted out in the final model using the variance inflation factor. A value of *p* < 0.05 was considered to be statistically significant. All statistical analyses were performed using the Statistical Package for Social Sciences (SPSS/Windows version 24, SPSS Inc., Chicago, IL, USA).

## 3. Results

Thirty-eight morbidly obese women, aged 46.3 ± 8.2 years, BMI 42.9 ± 3.6 kg/m^2^, were studied prior to RYGB and followed up at 1 year and 3 years after the intervention; 25.8% of them were menopausal. The patients’ characteristics are described in Table 1. As expected, weight, BMI and waist to hip ratio were dramatically decreased one year after surgery, with a slight but significant increase from year one to three. Indeed, 94.7% of patients had a BMI below 35 kg/m^2^ at year one, and 81.5% three years after surgery. The biochemical analysis showed normal plasma concentrations for calcium and phosphorus through the study. However, 25(OH)D_3_ values were low before surgery and their concentrations significantly decreased at one year and three years after surgery, in spite of recommended calcium and vitamin D supplementation. In particular, 36.8%, 28.9% and 71% of patients had a vitamin D deficiency at baseline, 12 months and at three years, respectively. PTH significantly rose, but remained below 10 pmol/L in 97% of patients at year one and 78.9% at year three.

Regarding body composition, a significant decrease in body fat was observed during the first year after surgery with a significant increase thereafter. Lean mass decreased during the first year and remained stable in the follow-up. 

Total BMD at femoral neck and lumbar spine decreased by 13.53 ± 5.42% and 6.03 ± 6.79% during the three-year follow-up, but these decreases were most important during the first year (Table 2). In spite of this reduction, *Z*-score values remained above those for women of the same age.

**Figure 1:**
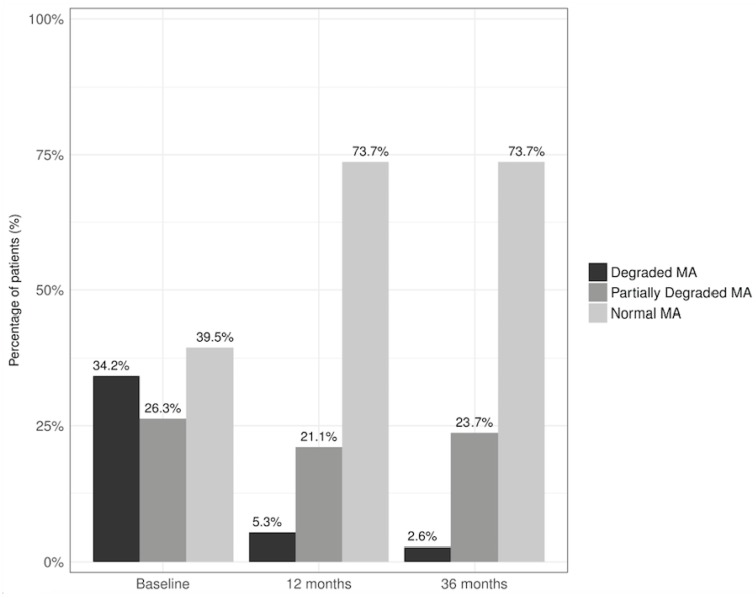
Percentage of patients with normal, degraded and partially degraded bone microarchitecture (MA) at baseline, 12 and 36 months after RYGB.

The evolution of TBS after bariatric surgery is shown in Table 2. All patients at baseline had a BMI greater than 35 kg/m^2^. Thus, values of Strict TBS were limited to the first and third years after surgery and their means were within normal ranges (1.431 ± 106 and 1.413 ± 85, respectively). Moreover, the percentage of patients with normal bone MA was of 73.7% and only 2.6% had degraded MA at the end of the study period (Figure 1). No significant differences were found in TBS values comparing menopausal to non-menopausal women (1.350 ± 130 vs. 1.410 ± 080, *p* = 0.115).

An analysis of the bone MA values in patients categorized with osteopenia and osteoporosis by T-score in the study was performed. At baseline, four patients (10.5%) presented with osteopenia. At three years, 13 (34.2%) patients had osteopenia and two (5.2%) had osteoporosis. From those 15 patients, one (6.6%) had degraded bone MA and five (33.3%) partially degraded bone MA and nine (60%) normal bone MA. 

**Figure 2:**
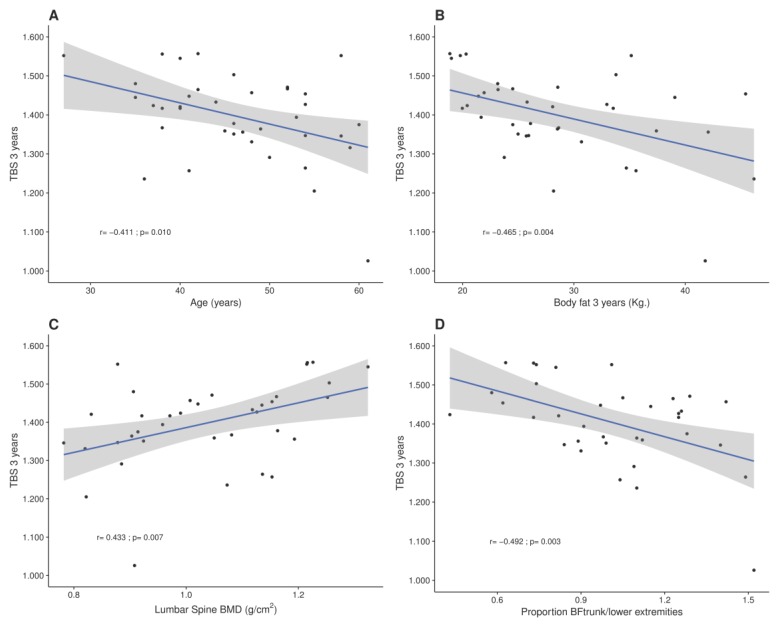
Correlations of TBS at three years after surgery with: (**A**) Age; (**B**) Body fat (BF); (**C**) LSBMD and (**D**) Body fat distribution trunk/versus lower extremities. Pearson correlation coefficients are included in each graph.

At three years, TBS correlated inversely with age (*r* = −0.41, *p* = 0.010), weight (*r* = −0.367, *p* = 0.025); body fat (*r* = −0.465, *p* = 0.004), and positively with LSBMD in the same time period (*r* = 0.433, *p* = 0.007) (Figure 2). Moreover, TBS at the end of the study correlated positively with weight loss (*r* = 0.54, *p* = 0.001), fat mass loss (*r* = 0.438, *p* = 0.007), and lean mass loss (*r* = 0.432, *p* = 0.008). In the multiple regression analysis, TBS values at three years remained associated with final body fat (*β* = −0.625, *p* = 0.031; *R*^2^ for the model 0.47) after adjusting for age, lean mass and vitamin D concentrations. 

In order to analyse for the possible interference of adipose tissue content around the waist in TBS determinations, adipose indexes were analysed. A negative association of TBS values at 3 years was found, with greater body fat distribution in the trunk versus legs (*r* = −0.482, *p* = 0.004), and trunk versus lower extremities (*r* = −0.492, *p* = 0.003) (Figure 2).

Major osteoporotic and hip fracture risk, calculated by FRAX^®^, with and without adjustment by TBS, at 3 years, showed no significant differences (3.19 ± 0.82% vs. 3.12 ± 1.34% and 0.11 ± 0.15% vs. 0.10 ± 0.13%; *p* = 0.324 and *p* = 0.66, respectively). Altogether, risk of facture was low and no patients experienced a bone fracture during the study.

## 4. Discussion

Bariatric surgery represents an effective treatment for severe obesity, with a rising number of procedures performed worldwide. However, there are still uncertainties about the effect of these surgeries on bone health and this is a growing matter for concern. TBS can offer an easy and non-invasive way to evaluate bone MA after bariatric surgery. Our study showed that mean TBS values were within normal ranges, three years after gastric bypass, reflecting good bone quality. 

The major determinants of bone strength are both BMD and skeletal MA. Prospective studies assessing BMD changes after bariatric surgery are numerous, with a few reviews and meta-analysis being published [3,4,5,6,7,8]. Most of the studies have been performed in small cohorts, after RYGB, and have observed a significant decrease in BMD in the first 12 months after surgery, mainly at the femoral neck, ranging from about 9–10% and a vertebral BMD decline of 3.6 to 8% [3,4,5]. It has been speculated that this decrease could be a normal adaptation to the decreased loading of the bone that would become stable after weight stabilization [3,6]. However, data on BMD descent after RYGB beyond the first postoperative year and in the long term are scarce [28,29]. The majority of cross-sectional and retrospective studies, comparing patients undergoing bariatric surgery to control groups matched by post-surgery BMI have reported a similar or greater bone mineral density following surgery at the femoral neck, lumbar spine and radius [9,10,11]. Moreover, studies reporting T and Z-scores have described normal scores in bariatric patients and a low rate of osteoporosis development [9,10,28]. In our study, in agreement with previous reports, a decrease of 13.5% in FN was observed and 6.03% at LS at three years evaluation, but Z-score values remained above those seen in women of the same age. 

However, BMD has shown to be a poor predictor of bone fractures, as patients with a normal BMD do experience fractures, suggesting the importance of bone quality. There are accurate methods for evaluating bone MA, such as histomorphometric analysis and micro-computed tomography of the transiliac crest bone biopsy, high-resolution peripheral quantitative computed tomography [30] and magnetic resonance imaging, but they are expensive and not routinely used outside clinical trials. In contrast, TBS is a non-invasive, non-expensive method to assess bone MA, based on the evaluation of pixel gray-level variations in the DXA image through the use of specific software. A main advantage is that TBS can be calculated from a previously done DXA exploration. A number of cross-sectional and prospective studies have shown an association between lumbar spine TBS and vertebral, hip and other types of osteoporotic fractures in postmenopausal women [20].

TBS was normal in our patients after bariatric surgery when they had a BMI within TBS validated ranges. In this sense, at the end of the study period, the percentage of patients with degraded MA was only 2.6%. To our knowledge, there is only one previous paper that has analyzed the changes in TBS after bariatric surgery. In this previous work, pre-menopausal women and men undergoing RYGB or SG were randomized to an intervention arm that included strict supplementation with vitamin D, calcium, protein and muscle exercise, versus a non-intervention group. TBS declined in both groups and then remained stable in the intervention group (−3.4% vs. −10.5%). The mean TBS was below 1.300 along all the points of the study [31]. The fact that the patients in this study had higher initial and final fat mass values and lower initial vitamin D compared to our cohort, and that the authors corrected TBS values by tissue thickness with a personal and not yet standardized formula, hinders the comparison with our findings.

In our study, TBS correlated negatively with BMI and specifically with body fat, this adiposity parameter being the main determinant in the multiple regression analysis. In agreement with our findings, some studies have described that this relationship differs from that observed with BMD. In this sense, BMI and lean mass are positively and more strongly related to BMD than fat mass [28,32] and particularly, the loss of lean mass after surgery is a key factor implicated with BMD decline. On the contrary, lumbar spine TBS correlates negatively with BMI and fat mass in most studies [33,34].

In order to interpret our results, we must consider that in an obese population, excessive soft tissue in the abdomen may reduce the TBS estimate, so pre-surgical values can be altered by artifacts. Indeed, our data showed a negative correlation between TBS values and greater body fat distribution in trunk versus legs. Similar findings were described in the Romagnoli study [34] that evaluated TBS in overweight/obese men, concluding that waist circumference, instead of BMI, should probably be taken into account when assessing TBS. Nevertheless, at the final evaluation, patients’ BMIs were below 35 kg/m^2^ and in this clinical situation, body fat provokes little image noise, thus making TBS accurate.

Fracture risk after bariatric surgery is still a matter of debate. In some studies fracture risk doubled after RYGB, compared to the general population [12] and also when compared to adjustable gastric banding [16]. In other studies, the increased adjusted relative risk was observed only after malabsorptive surgeries at traditional osteoporotic sites [14] or in non-traditional osteoporotic sites [13]. However, the only retrospective study using weight-matched controls, performed by Lahmohamed, including 2079 patients, with a mean follow-up of 2 years, did not observe a change in fracture risk following surgery [15]. A limitation of previous studies is their retrospective design and the difficulties in selecting appropriate controls. To date, no prospective fracture data exists. In our study, none of the patients presented with bone fractures, but our follow–up was short.

The FRAX^®^ algorithm is routinely used to predict the 10-year probability of fracture risk. TBS is currently used to adjust FRAX values. In fact, it is estimated that TBS allows the reclassification of the fracture risk by 2.5% with a net improvement of 7.6% [35]. In our population, the fracture risk, assessed by FRAX, when adjusted for TBS, was unchanged, as expected, when TBS was within a normal range, as was the case. Of note, the observed major osteoporotic fracture value was 3.12% and hip fracture risk was 0.11%, which are low percentages, since the current guidelines from the National Osteoporosis Foundation recommends medical treatment in patients with osteopenia, only when the 10-year probability of major osteoporotic fracture is equal to 20% or the hip fracture is 3% [36].

Another interesting finding from our study is that vitamin D concentrations did not correlate with variations in BMD or TBS scores. A decrease in vitamin D concentrations was observed over time, that would explain the subsequent rise in PTH plasma levels. This fact can be attributed to lack of treatment compliance or insufficient dosing. Although in the short term vitamin D does not seem to have a direct effect on BMD changes, there is a need to individualize and intensify its replacement in order to minimize the risk of bone deterioration. 

Our study has several limitations. The sample size is relatively small, the follow-up was short and all participants were female, which limits the generalization of the findings. Although some TBS studies have been performed in an obese population, there is a current lack of standardized data in obesity and the use of TBS in subjects with BMI over 35 kg/m^2^ has not been yet validated. Baseline TBS in our population was therefore not interpretable and we were not able to analyze changes in TBS during the first year after surgery when the maximum BMD loss occurs. We therefore cannot exclude a deterioration of bone MA during this initial period. However, this is an intrinsic limitation of the technique and the manufacturers are working at present on formulas for BMI correction that are not yet available. On the other hand, the fact that TBS is only available for lumbar spine and is not useful for the evaluation of appendicular sites, has to be taken into account. Other factors that influence bone health, such as exercise and smoking, were not recorded. Moreover, it has been suggested that leptin and adiponectin may act as mediators between surgery and the BMD changes but we have not analyzed the influence of adipokines in TBS changes.

However, in spite of the technique limitations, TBS offers a non-invasive method to assess bone MA. Our findings would indicate that women undergoing RYGB have a preserved bone MA in the mid-term. Further analyses in the long-term are required to better assess the accuracy of TBS in obese individuals and how this impacts on fracture prediction. 

## Figures and Tables

**Table 1:** Demographic, clinical and biochemical characteristics of study population.

Variables	Basal	12 Months after Surgery	36 Months after Surgery
Age (years)	46.39 ± 8.28		
Weight (kg)	109.25 ± 10.79	72.62 ± 9.50 *	77.30 ± 11.3 **
BMI (kg/m^2^)	42.91 ± 3.62	28.50 ± 3.72 *	30.40 ± 4.46 **
Waist to Hip Ratio	0.87 ± 0.08	0.83 ± 0.06 *	0.85 ± 0.06 **
DXA FM (kg)	46.92 ± 6.19	23.26 ± 5.95 *	28.68 ± 7.66 **
DXA LM (kg)	54.97 ± 4.83	47.22 ± 4.53 *	46.50 ± 5.42
DXA BF (%)	44.90 ± 3.14	31.61 ± 4.84 *	36.72 ± 5.10 **
Calcium (mmol/L)	2.31 {0.13}	2.40 {0.16}	2.31 {0.20} **
Phosphorus (mmol/L)	1.10 ± 0.16	1.30 ± 0.14 *	1.14 ± 0.23 **
PTH (pmol/L)	4.10 {2.45}	5.6 {3.05} *	6.9 {5.85} **
25(OH)D_3_ (nmol/L)	57.4 {47.2}	61.4 {41.5} *	34.1 {21.0} **

All data are expressed as mean ± standard deviation (SD) and median {interquartile range}. BMI: body mass index; FM: fat mass; LM: lean mass; BF: body fat; PTH: parathyroid hormone; 25(OH)D_3_: 25-hydroxyvitamin D; IGF-I: insulin-like growth factor-I; * *p* < 0.05 comparing 12 months to baseline values; ** *p* < 0.05 comparing 3 year to 12 month values.

**Table 2:** Bone measurements before and after gastric bypass.

Variables	Basal	12 Months after Surgery	36 Months after Surgery
Femoral neck			
BMD (g/cm^2^)	1.10 ± 0.12	0.98 ± 0.12 *	0.94 ± 0.11 **
*T*-Score	1.01 ± 1.06	0.06 ± 1.05 *	0.06 ± 0.92
*Z*-Score	1.49 ± 0.78	0.58 ± 1.02 *	0.56 ± 0.86
Lumbar Spine			
BMD (g/cm^2^)	1.09 ± 1.33	1.06 ± 0.14 *	1.04 ± 0.14 **
*T*-Score	0.65 ± 1.30	0.21 ± 1.28 *	−0.38 ± 1.30 **
*Z*-Score	1.05 ± 1.13	0.74 ± 1.10 *	0.40 ± 1.23 **
AllTBSvalues	1.271 ± 0.183	1.431 ± 0.104 *	1.396 ± 0.109
Strict TBS values	-	1.431 ± 0.106 *	1.414 ± 0.086

All data are expressed as mean ± standard deviation (SD). BMD: Bone Mineral Density; TBS: trabecular bone score; * *p* < 0.05 comparing 12 month to baseline values; ** *p* < 0.05 comparing 3 year to 12 month values.
